# A Novel Electronic Nose as Adaptable Device to Judge Microbiological Quality and Safety in Foodstuff

**DOI:** 10.1155/2014/529519

**Published:** 2014-03-24

**Authors:** V. Sberveglieri, E. Nunez Carmona, Elisabetta Comini, Andrea Ponzoni, Dario Zappa, Onofrio Pirrotta, A. Pulvirenti

**Affiliations:** ^1^Department of Life Sciences, University of Modena and Reggio Emilia, Via Amendola, 42122 Reggio Emilia, Italy; ^2^CNR-INO Sensor Lab, Via Valotti 9, 25133 Brescia, Italy; ^3^CNR IBF, Via Ugo La Malfa 153, 90146 Palermo, Italy; ^4^Department of Information Engineering, University of Brescia, Via Valotti, 25133 Brescia, Italy; ^5^University of Modena and Reggio Emilia, DISMI, Via Amendola, 42122 Reggio Emilia, Italy

## Abstract

This paper presents different applications, in various foodstuffs, by a novel electronic nose (EN) based on a mixed metal oxide sensors array composed of thin films as well as nanowires. The electronic nose used for this work has been done, starting from the commercial model EOS835 produced by SACMI Scarl. The SENSOR Lab (CNR-INO, Brescia) has produced both typologies of sensors, classical MOX and the new technologies with nanowire. The aim of this work was to test and to illustrate the broad spectrum of potential uses of the EN technique in food quality control and microbial contamination diagnosis. The EN technique was coupled with classical microbiological and chemical techniques, like gas chromatography with mass spectroscopy (GC-MS) with SPME technique. Three different scenarios are presented: (a) detection of indigenous mould in green coffee beans, (b) selection of microbiological spoilage of Lactic Acid Bacteria (LAB), and (c) monitoring of potable water. In each case, the novel EN was able to identify the spoiled product by means of the alterations in the pattern of volatile organic compounds (VOCs), reconstructed by principal component analysis (PCA) of the sensor responses. The achieved results strongly encourage the use of EN in industrial laboratories. Finally, recent trends and future directions are illustrated.

## 1. Introduction

Aroma is one of the most significant parameters of foods from the sensory point of view. The characteristic flavour of VOCs, so called fingerprint, may offer information about safety and quality of food, performing sometimes as an indicator of process mistakes as well [1].

Indeed, some volatile compounds can be originated from biochemical processes of food, as a consequence of technological food chain or product storage.

Unwanted smell, so-called off-flavour, may involve substances originating from the metabolism of spoilage microorganisms, bacteria, and fungi that adulterate naturally or unintentionally the products before or during its production [2].

In the last decade, electronic noses (EN) have become very popular as monitoring tools in evaluating food quality and safety [3].

In this paper, three important applications of EN in food control were examined, concealing three relevant issues in the food field of food quality and control.

Another main target of this work was to illustrate the broad spectrum of potential uses of sensor technology in this field and to show the potential of the new Nanowire technology.

At SENSOR laboratory, the studies on chemical sensors started at 1988 with the improvement of thin films and then of a new technique for the planning of thin films with an extremely porous structure [4].

In 2001 after the first publications demonstrating the possibility of preparing metal oxide in forms of nanowires and nanobelt, SENSOR demonstrates the ability of metal oxide nanowires in detecting variety of chemical species [5].

It is well know from six decades that metal oxide electrical property depends on the surrounding atmosphere.

**Figure 1:**
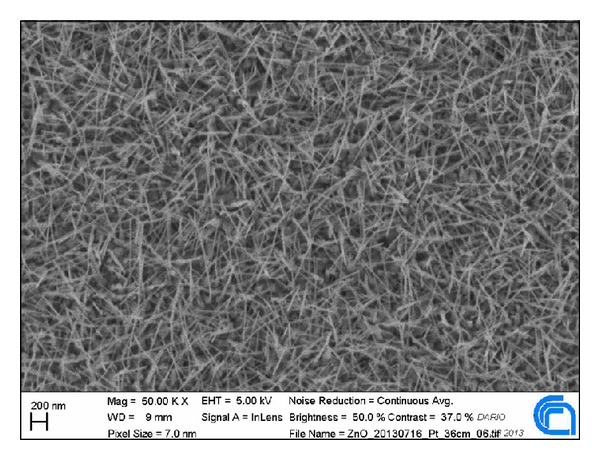
ZnO nanowires SEM picture, at 50 k magnification.

Quasi-one-dimensional metal oxide nanostructures (Figure 1) have several advantages with respect to thin and thick film counterparts such as large surface-to-volume ratio, lateral dimensions comparable to the surface charge region, and superior stability when in the single crystal structure [6].

**Figure 2:**
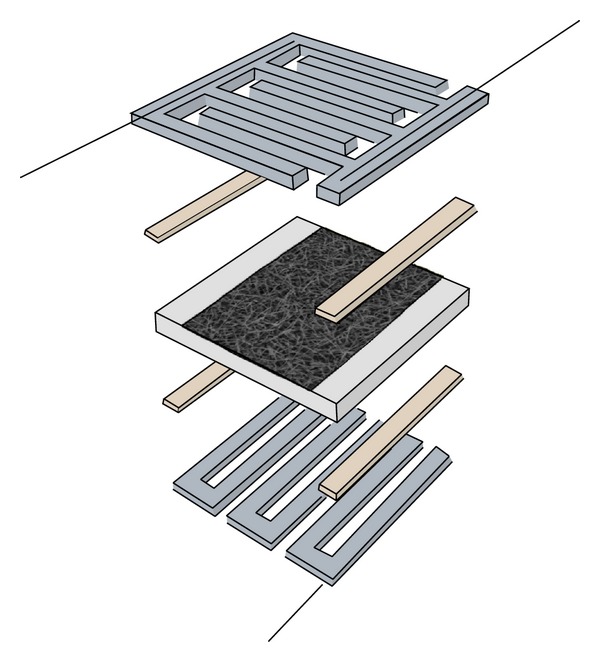
Sketch of the conductometric device. Alumina substrate is white colored in the middle, TiW pads are in brown, and platinum heater and contacts are in metallic gray.

Single crystal nanostructures (Figure 2) of tin oxides have been fabricated and characterized as sensing materials to be implemented in an electronic nose.

These nanowires exhibit remarkable crystalline quality and a very high length-to-width ratio, resulting in enhanced sensing performances as well as long-term stability for sustained operation [7].

## 2. Materials and Methods

- All the coffee, produced and consumed, belongs to the genus* Coffea* that focuses principally two species:* C. arabica* and* C. canephora*. Those species generally mature in the equatorial zone, where the environment conditions of humidity, temperature, wind, rains, and altitude permit the harvest of many different varieties, each one with particular requirements.

Most of the producers are developing countries, so the storage conditions and the methods used for harvesting and shipping of green coffee beans depend on the production country.

For this reason, it can be easily contaminated by mould throughout the food chain.

Usually the contamination appears due to moulds belonging to the genus* Aspergillus*.

The selection of the raw material occurs in the early stages of the processing chain by visual inspection. Parameters that determinate the quality of green coffee beans are shape, colour, and size.

Frequently the raw material is already contaminated when this selection occurs and thus the detection of the contamination became very difficult.

Rose-Bengal Chloramphenicol Agar (Sigma-Aldrich Chemical Co., St. Louis, MO, USA) was used, as a selective medium for the enumeration of yeast and moulds from a wide variety of distinctive food matrix.

The Chloramphenicol works as suppressor of bacteria growth, although the Rose-Bengal acts as a limiting agent of the mould growth. In order to assist the enumeration of small colonies, the media have neutral pH. The same kind of medium was used to perform all the analysis.

In the case of GC-MS-SPME and EN analysis, 5 mL of medium was placed into sterile 20 mL chromatographic vial and left to solidify.

All the samples, for microbiological analysis, CG-MS-SPME and EN were prepared on the first day of the analysis, taking this day as zero-time. Once prepared, all the samples were stored and incubated under the same conditions during all the duration of analysis, namely, 28°C for a total of 7 days.

During the 7 days of the experiment, analysis was done at zero-time, inoculation day, at T3 (3 days after the inoculation), at T4 (4 days after the inoculation), at T6 (6 days after the inoculation), and at T7 (7 days after the inoculation).

For each day of GC-MS analysis, 10 vials of every type of samples were prepared (control, Honduras, Indonesia, and India): one of each type was used for the GC-MS-SPME analysis, while the other 36 for the EN analysis.

For each day of analysis, the vials were crimped and incubated in an oven thermostatically regulated at 40°C for 15 minutes, to create the headspace equilibrium.

In order to extract the volatile compound from the samples, a DVB/carboxen/PDMS stable flex (50/30 *μ*m) (Supelco Co., Bellefonte, PA, USA) SPME fiber was used.

To furnish the adsorption of volatile compounds, the SPME fiber was exposed to the headspace of the vials for 15 minutes at room temperature. For desorption of the compounds, the fiber was placed in the injector of the heated GC for 6 min.

The ramping of temperature in the column was performed in the following way: 60°C for 2 min to 100°C at 5C°/min, followed by a rise from 100°C to 240°C at 5°C/min and then this temperature was kept for 5 min. Chromatographic analysis was accomplished using a HP 6890 series GC system, 5973 mass selective detector with a DB-WAX capillary column. The injection was verified in splitless mode at 240°C using helium as gas carrier with a setting flow of 1.5 mL/min.

For the electronic nose the sample headspace (4 mL) was then extracted from the vial in static headspace path and injected into the carried flow (speed 4 mL min) through a properly modified gas chromatography injector (with the connection tube to the EN kept at 40°C to prevent any condensation).

A synthetic chromatographic air with a continuous flow rate of 10 mL/min was used to recover the sensor baseline, resulting in a recovery time of 28 min.

(b) In the case of LAB analysis, the samples were treated using spoilage lactic acid microflora isolated from the chicken meat. The used procedure for sampling method was conducted as it is described below. Under sterile conditions, 10 g of chopped chicken meat was placed in a stomacher bag with 90 mL of sterile physiological solution and shacked off in Lab Blender Stomacher 400 (Type BA 7021 Seward, London) for 1 min at normal speed (200 paddles/min).

One mL of the supernatant was inoculated in Man, Rogosa and Sharpe Agar medium (MRSA) (OXOID) [8] Petri dish following the inclusion method. Once the first layer was solid, a second layer was added in order to create the microaerobic environment conditions for the LAB growth. MRSA medium was considered to be supporting the growth of lactobacilli. The plates, inside jar with the gas generating kit (OXOID), were incubated during 48 hours at 30°C.

Then, the 48-hour 3 colonies were randomly picked up and inoculated in a MRS liquid tube in order to obtain liquid cultures. The 3 typologies of tubes were incubated for 48 hours at 30°C.

The samples for EN and GC-MS analysis were prepared using the same procedure. Sterilized chromatographic vials (20 mL) containing 2 mL of MRSA media were inoculated independently with 100 *μ*L of the number 3 of McFarland standards of the 3 kinds of cultures prepared before. These standards are used as a reference to adjust the turbidity of bacterial suspensions in order to have a number of bacteria within a given range. Number 3 of McFarland standard matches a bacterial concentration of 9 × 10^8^ CFU/mL.

Analysis with EN and GC-MS was done at 0 time, inoculation day, and 24 hours later a second cycle of GC-MS was performed to make a control of the head space changes at the end of the EN analysis.

(c) Regarding the incidence of coliforms, an aliquot of water from wc and a well was dispersed in 2 Petri dishes with Violet Red Bile Agar (VRBA) (OXOID) [9]. VRBA is a selective medium used for the detection and enumeration of coliform bacteria in water and other food dairy products. The goal was to isolate the single colonies that were used later to inoculate liquid tubes of Brilliant Green Bile medium (OXOID) [10] to obtain pure liquid cultures. Brilliant Green Bile medium is a modification of MacConkey's liquid medium for the isolation of Enterobacteriaceae and has been formulated to obtain maximum recovery of bacteria of the coli-aerogenes group, while inhibiting most gram-positive bacteria.

For the 2 kinds of samples the followed procedure was the same. Once the Petri dishes were inoculated, they were conserved at room temperature for 2 days. Then, single colonies were selected and inoculated in liquid tubes of Brilliant Green Bile media and incubated for 24 hours at the optimal growth temperature for coliforms 35°C.

After 24 hours, the turbidity of the tubes was evident, and it was adjusted (diluted, using sterile Brilliant Green Bile medium) until the turbidity was the same as the number 3 of the McFarland standards.

Samples GC-MS-SPME and EN were prepared on the first day of the analysis taking this day like 0 time. Once prepared, all the samples were stored and incubate under the same conditions during all the lasting of analysis, namely, 35°C for a total of 24 hours.

The analyses were done the same day of inoculation, and a second cycle of GC-MS was made 24 hours later. In this case, the samples were not crimped and the ensemble was cover with aluminium foil in order to keep vial and cap combined to preserve the sterility inside and, at the same time, afford the aerobic condition for the bacterial growth.

Principal component analysis (PCA) performed explorative data analysis. Data were processed by EDA software, at-home-written software developed in MATLAB at SENSOR laboratory [11].

Exploratory data analysis (EDA) is a fundamental step in the data analysis cycle (the cycle consists of data acquisition, data preprocessing, exploratory data analysis, and classification).

The aims of explorative analysis are as follows: maximize insight into a data set, uncover underlying structure, extract important features, and detect outliers. The most valuable outcome of EDA is to check for prior assumptions and determine optimal experimental settings.

## 3. Results and Discussion

- After 7 days of incubation (classical microbiological analysis) the differences among the inoculated plates were manifested. Each sample corresponds to one of the 3 different provenances of coffee selected for the experiments. It's perfectly shown the differences in number and typology of colonies between the samples.

These results suggest that coffee from different provenances have qualitative and quantitative difference in indigenous contamination.

Results obtained with GC-MS showed to be in perfect correlation with those obtained with the other two techniques.

These differences are both qualitative and quantitative, showing in all cases, except the control, compounds that corresponded with the metabolites produced by moulds during their growth.

In particular, it can be highlighted the formation of carbon dioxide, ethanol, and compounds belonging to chemical indole group [12].

**Figure 3:**
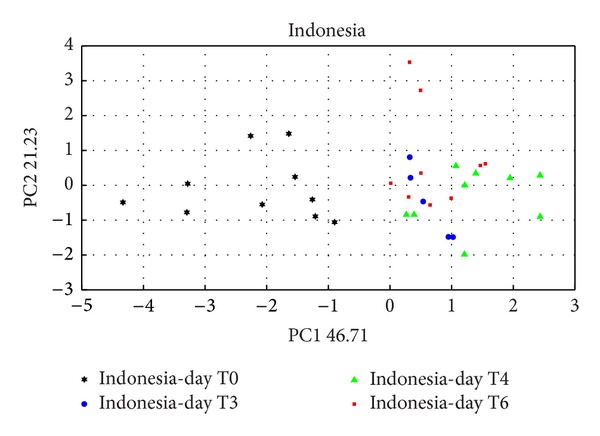
PCA score plot about Indonesia green coffee beans during 6 days of analysis (T0 to T6).

**Figure 4:**
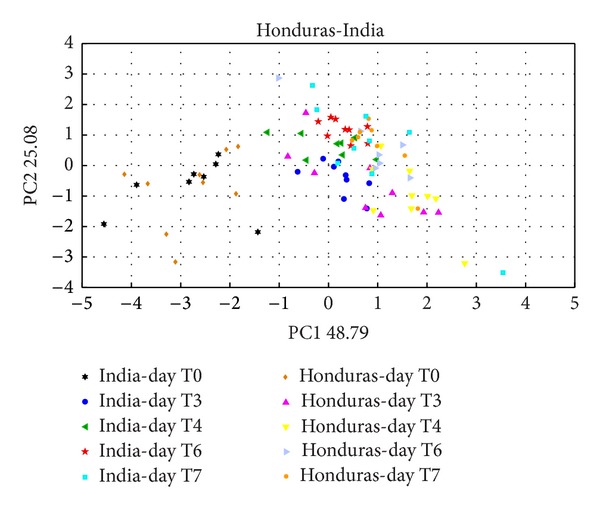
PCA score plot about the comparison of two different origins, Honduras and India green coffee beans (T0 to T7).

In the figures (Figures 3 and 4) are shown the data related to Indonesia, Honduras, and India, from day T0 (day of the sample preparation) to days T6 and T7 (six and seven days after sample preparation).

It is clearly visible a separation in the PC1 axis among the days of analysis, showing development of the growth of the moulds that in both case has more statistical significance.

In the previous literature [12], all the results were referred to an array of 6 MOX thin film sensors that provide a real individualization of the samples only at day T6.

The new array composed of 4 MOX [7] thin film sensors and other 2 made up with MOX nanowire technology; the differences between samples are evident already at T3, halving the response time and hence increasing the instrument threshold.

**Figure 5:**
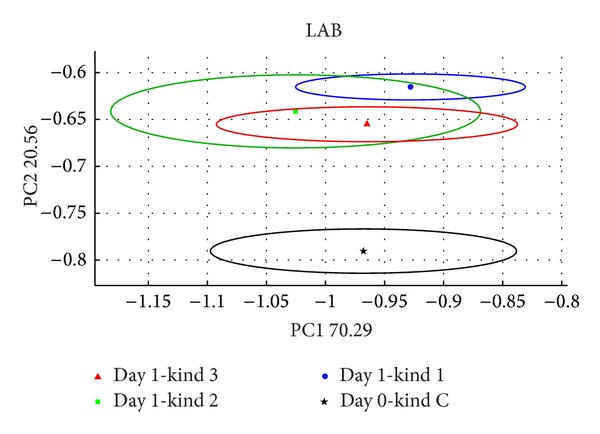
PCA score plot about control in a separate cluster and 3 different kinds of LAB. In the blue circle the kind 1, in the red circle the kind 3, in green circle the kind 2, and in black circle in the bottom the control sample.

(b) In Figure 5 are showed the result from the PCA analysis of LAB. It can be separate 2 principal clusters. One denotes to the control (black circle), and the second one is formed for 3 typologies, samples belonging to different colonies.

The creation of cluster like this one can be due also because the VOCs that the EN is able to detect were present in the total pathway of the LAB and to the similarity of the colonies because of the same provenience of the samples, all them indigenous contaminated of the chicken meat.

Regarding the kind 1 (blue circles), samples belonging to colony 1 it worth to think that can be bacteria from the same group but perhaps to different species so it will explain that some of the samples start to move away from the general cluster.

**Figure 6:**
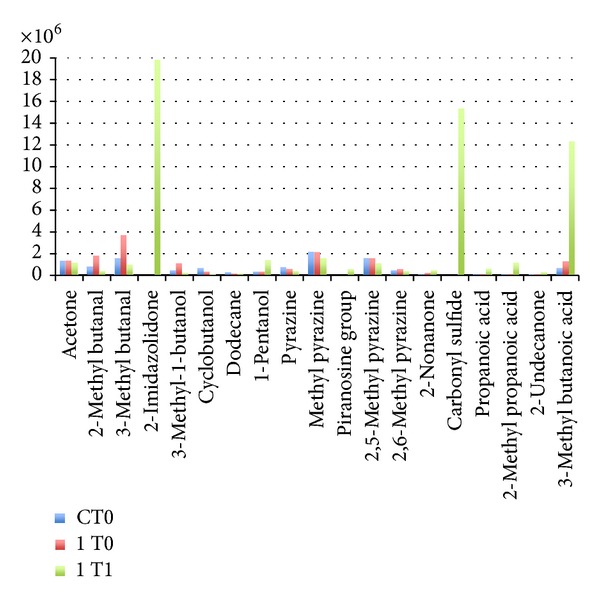
Histogram obtained from GC-MS, with SPME techniques about VOCs produced by control and one kind of LAB.

The result obtained in LAB, with GC-MS (Figure 6), case seems to be in perfect correlation with those obtained with the EN. Actually, there are evident qualitative and quantitative differences within the components of samples of the same group.

**Figure 7:**
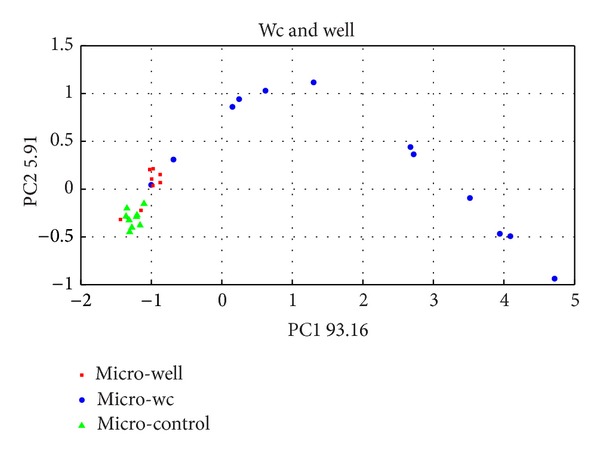
PCA score plot analysis of microorganism isolated from wc and well (pozzo).

(c) In the case of PCA (Figure 7) from the EN analysis of coliforms, a separate cluster formed by the samples of the bacteria isolate from the well and those belonging to wc can be observed. Very interesting results came out concerning the samples proceeded from the isolate of the wc. In this case the different measures form a curve perfectly correlated with the time in which the measure was done, showing a kind of curve from bacterial growth. This growth correspond to 20 hours of EN analysis and the curve showed by the PCA analysis can be compared with the first steps of the typical logarithmic growth curve of this species of microorganism.

**Figure 8:**
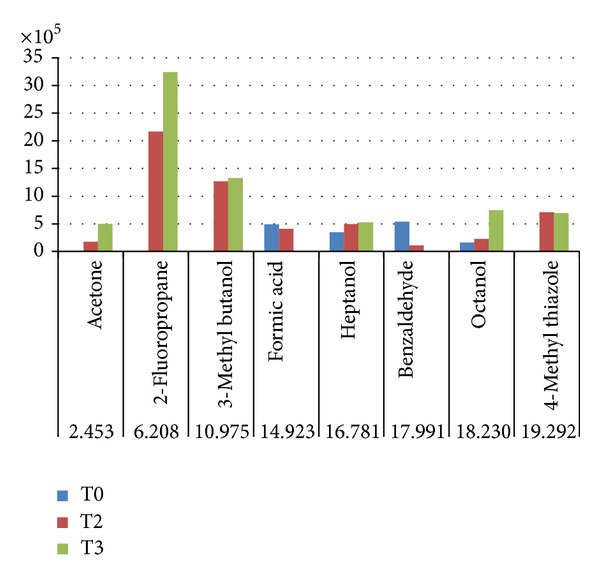
Histogram obtained from GC-MS, with SPME techniques about VOCs produced by wc samples (T0 to T2).

In the case of coliforms, it can be observed, in the results obtained with GC-MS (Figure 8), an increase of the acid compounds like benzoic, hexanoic acid, 3 methyl butanol, and also some compounds with an alcohol group like nonanol and acetone.

In some cases, EN is able to detect differences between samples while the GC-MS is not able to reveal a production of metabolites created by the growing bacteria. That is owing to the consumption, by the bacteria, of some metabolites present in the medium, making the EN respond in different way and revealing differences between the samples.

## 4. Conclusions

In this work, some significant applications of an electronic nose, based partially on metal oxide nanowires technology, to microbiological food quality control have been review. Literature review has been accompanied by some significant case studies previously presented in this field by the same authors, in order to provide the reader with an enhanced perceptiveness of the EN application.

All the described case studies showed promising results, thus confirming that our EN could represent a rapid mean for controlling and improving the microbiological quality of food.

Keeping in mind advantages and limitations, EN does not allow replacing human panels or analytical techniques yet. Their ability to smell odours rather than detecting and quantifying specific volatiles (VOCs) can still be improved. However, they can be used in parallel to those techniques, or ever considered as valuable alternative. Current methodology involves conventional technique such as classical microbiology, visual techniques, or molecular techniques and requires in most of cases a big amount of time, not always available.

This work attests that the electronic nose, once trained, is a potential and useful (rapid and economic) tool for the early detection of microbial grows. A kind of sensor technology like a novel EN provides a faster response of the detection of contaminations in food matrix than the conventional techniques (also compared with the commercial EN equipped only with traditional MOX sensors).

In some cases the novel EN is able to anticipate the detection in a few days with respect to the commercial one [12]. It is possible thanks to the dimensional structure of the sensors that give more surfaces for contact with the VOC's map.
